# Editorial: What can Neuroscience Learn from Contemplative Practices?

**DOI:** 10.3389/fpsyg.2015.01731

**Published:** 2015-11-10

**Authors:** Zoran Josipovic, Bernard J. Baars

**Affiliations:** ^1^Psychology Department, New York UniversityNew York, NY, USA; ^2^The Neurosciences InstituteSan Diego, CA, USA

**Keywords:** meditation, mindfulness, neural correlates of consciousness, structural plasticity, functional plasticity, contemplative practice, cultural issues

Contemplative practices like meditation and mindfulness have recently gained increased acceptance in science and clinical practice, although a number of issues related to their phenomenology and to experimental designs still remain (Dahl et al., 2015).

## Signs of progress

Significant progress has been made in the area of the neuroimaging of meditation and mindfulness, leading to increased understanding of the neural mechanisms underlying different techniques and stages of meditation (Lutz et al., 2008; Travis and Shear, 2010; Vago and Silbersweig, 2012; Craigmyle, 2013; Josipovic, 2014; Tang et al., 2015a). Results point to increased flexibility and efficiency of the brain's networks, and to enhanced functional and structural integration among their nodes (Braboszcz et al., 2013; Luders et al., 2013; Tang et al., 2015a).

The effects of meditation and mindfulness on physiological measures have been researched extensively. Although some of these findings have been challenged over the years, others, such as cortisol level decrease, enhanced immune response, decreased chronic pain, etc. have held. Recent findings of epigenetic changes due to relaxation response (Bhasin et al., 2013), focused attention meditation (Jacobs et al., 2013), and mindfulness (Carlson et al., 2014), may have significant clinical implications. Changes in structural plasticity in the brain due to both long-term (Luders et al., 2013; Kurth et al., 2015) and short term meditation training (Hölzel et al., 2011; Tang et al., 2012), provide further, though indirect, evidence of epigenetic effects.

Increasingly, studies point to beneficial effects of meditation and mindfulness on cognition, affect, and social behavior, though the findings can be contradictory at times and the effect sizes small (Braboszcz et al., 2013; Leonard et al., 2013; Ben-Soussan et al., 2014; for review see Dahl et al., 2015; Tang et al., 2015a). The effects on attentional networks have been seen most clearly in long-term practitioners, or after longer (3 month) retreats, with possible differential effects on alerting, orienting and executive attention networks at different stages of practice (Chiesa et al., 2011). Effects on the working memory, conflict monitoring and response inhibition, and the increased activation of related prefrontal areas, have been proposed as the top-down mechanism mediating the effects of mindfulness on emotion regulation (Vago and Silbersweig, 2012; Tang et al., 2015b). The overall pattern that emerges is one of initial reliance on the effortful top-down control that gradually shifts, with an acquisition of expertise, to a more effortless implicit bottom-up regulation. Understanding how different meditation techniques affect the sense of self, whether deconstructing or reconstructing it, may prove to be the key in understanding the more lasting effects of meditation (Austin, 2013; Tang and Tang, 2013; Dahl et al., 2015).

The validity of introspection has been a perennial issues for contemplative traditions. Though experienced meditation practitioners may be more accurate in reporting their experiences than average subjects (Lutz et al., 2007), the choice of contents reported, and the manner of reporting them, are often influenced by the language and beliefs of the tradition subjects belong to. Thus training research subjects in the art of phenomenological epoche may be necessary.

Meditation and mindfulness practices can also generate intense and unusual experiences and altered states of consciousness, such as states of reduced phenomenal content, or absorptions (Lutz et al., 2007). These are akin to states of deep relaxation, or even deep sleep, but without actually sleeping. They can have other unusual features, such as alterations in the sense of time and space (Berkovich-Ohana et al., 2013), or spontaneous perceptions of light patterns (Lindahl et al., 2014). An intuitive, but arguable, idea is that most of these states should lead to global decreases in cortical activity (Hinterberger et al., 2014; Berkovich-Ohana et al., 2015), or at least to decreases in the areas related to spontaneous thinking (Brewer et al., 2011). Perhaps even more interesting are the states of reduced phenomenal content accompanied by increased awareness. The subjects report experiencing their consciousness as being relatively “pure,” an awareness without a content. These may emerge at first as brief interruptions in one's usual stream of consciousness during meditation (Baars, 2013), then get progressively more stabilized and longer lasting, until eventually one can find the “pure” nondual awareness present as a background context of all one's experiences, including dreaming and deep sleep (Travis et al., 2002; Ferrarelli et al., 2013; Josipovic, 2014; Thompson, 2014). Baars (2013) discusses some possible ways of approaching the research of these states. Expanding the neuroscience view of consciousness to include certain perspectives found in contemplative traditions may help to resolve some of the current impasses in debates about the neural correlates of consciousness (Block, 2007; Cohen and Dennett, 2011; Lau and Rosenthal, 2011; Baars et al., 2013).

## Ongoing challenges

One of the most challenging issues for meditation studies is the lack of accurate indices of subjects' experience during meditation, independent from subjects' reports. Several neurophysiological measures have been proposed over the years, however, it is not likely that any single measure can adequately capture the complexity of meditation experience (Davis and Vago, 2014). An interesting recent development are the attempts to obtain experience sampling data and provide neurofeedback via wearable devices and cell phones, as an adjunct to meditation training. As Brandmeyer and Delorme (2013) point out such attempts still await further scientific developments in sensor technology.

Several persistent methodological issues have plagued meditation research studies since the early days (Nash and Newberg, 2013; Dahl et al., 2015; Tang et al., 2015a). Reliance on self-report measures with inadequate controls for placebo effect and demand characteristic can make the results questionable. Obtaining neuroimaging and other physiological measures that parametrically co-vary with self-report measures can remedy this and facilitate assessing the meaning of results. However, more direct systematic replications of results are needed. Replication attempts can be compromised when subjects or researchers in the original and replication studies belong to schools of contemplative practice that define the same meditation differently. Largely due to funding limitations, most meditation studies are still of a pilot kind, with a small number of self-selected subjects, utilizing within-subject or cross-sectional designs, and often with inadequate control groups for the placebo effect. Large scale, randomized, longitudinal studies with active control groups can overcome some of these shortcomings (Tang et al., 2015a).

## Difficulty in relating to traditional views

The extraordinary multiplicity of meditation techniques and seemingly contradictory effects they produce pose a significant challenge for researchers. While the current research-oriented taxonomies have addressed this problem through categorizing meditation techniques into two or three major styles (Lutz et al., 2008; Josipovic, 2010; Travis and Shear, 2010), further optimizing is needed for such taxonomies to be more accurate and comprehensive (Nash and Newberg, 2013; Newberg, 2014; Dahl et al., 2015).

Contrary to popular “one-size-fits-all” approaches and advertisements, different meditations can have differential effects depending on one's psychological and physical makeup, and on the stage of one's practice. Adverse effects of meditation and mindfulness, which are often discounted in traditional contexts, can be significant and are only recently being studied in a systematic way (Garland et al., 2015).

Taking meditation out of its cultural, religious, and philosophical contexts may miss the influences that these contexts can have on the observed results. Future research will need to include spiritual and religious motivations, ethical concerns, as well as interpersonal and cultural contexts (Nash and Newberg, 2013; Dahl et al., 2015). The views on the overall goal of contemplative practice can be diametrically opposed both between different traditions and within the sects of the same tradition, and can significantly influence how individuals practice, which experiences they cultivate, and which ones get selected for research (Davis and Vago, 2014).

Science alone, in its present form, may not be able to answer ontological and metaphysical questions about the nature of consciousness that are the focus of contemplative traditions (Delorme et al., 2013). New scientific methods, and a more integrated approach that combines humanities and sciences, may be necessary to encompass the vastness of human experience that meditations can lead to.

### Conflict of interest statement

The authors declare that the research was conducted in the absence of any commercial or financial relationships that could be construed as a potential conflict of interest.
